# Prevalence of at-risk genotypes for genotoxic effects decreases with age in a randomly selected population in Flanders: a cross sectional study

**DOI:** 10.1186/1476-069X-10-85

**Published:** 2011-10-05

**Authors:** Hans B Ketelslegers, Roger WL Godschalk, Ralph WH Gottschalk, Ad M Knaapen, Gudrun Koppen, Greet Schoeters, Willy F Baeyens, Vera Nelen, Joep PM Geraedts, Joost HM van Delft, Jos CS Kleinjans, Nicolas A van Larebeke

**Affiliations:** 1 Department of Health Risk Analysis and Toxicology, Maastricht University, Universiteitssingel 50, 6229ER Maastricht, The Netherlands; 2 Environmental toxicology, Flemish Institute of Technological Research (VITO), Boeretang 200, 2400 Mol, Belgium; 3 Analytical and Environmental Chemistry Department, ANCH, Vrije Universiteit Brussel, Pleinlaan 2, 1050 Brussels, Belgium; 4 Provincial Institute of Hygiene, Kronenburgstraat 45, 2000 Antwerp, Belgium; 5 Department of Genetics & Cell Biology, Research Institute GROW, Faculty of Health, Medicine & Life Sciences, Maastricht University, PO Box 5800, 6202 AZ Maastricht, The Netherlands; 6 Study Centre for Carcinogenesis and Primary Prevention of Cancer, Department of Radiotherapy and Experimental Cancerology, Ghent University Hospital, De Pintelaan 185 3K3, 9000 Ghent, Belgium

## Abstract

**Background:**

We hypothesized that in Flanders (Belgium), the prevalence of at-risk genotypes for genotoxic effects decreases with age due to morbidity and mortality resulting from chronic diseases. Rather than polymorphisms in single genes, the interaction of multiple genetic polymorphisms in low penetrance genes involved in genotoxic effects might be of relevance.

**Methods:**

Genotyping was performed on 399 randomly selected adults (aged 50-65) and on 442 randomly selected adolescents. Based on their involvement in processes relevant to genotoxicity, 28 low penetrance polymorphisms affecting the phenotype in 19 genes were selected (xenobiotic metabolism, oxidative stress defense and DNA repair, respectively 13, 6 and 9 polymorphisms). Polymorphisms which, based on available literature, could not clearly be categorized *a priori *as leading to an 'increased risk' or a 'protective effect' were excluded.

**Results:**

The mean number of risk alleles for all investigated polymorphisms was found to be lower in the 'elderly' (17.0 **± **2.9) than the 'adolescent' (17.6 **± **3.1) subpopulation (*P *= 0.002). These results were not affected by gender nor smoking. The prevalence of a high (> 17 = median) number of risk alleles was less frequent in the 'elderly' (40.6%) than the 'adolescent' (51.4%) subpopulation (*P *= 0.002). In particular for phase II enzymes, the mean number of risk alleles was lower in the 'elderly' (4.3 ± 1.6 ) than the 'adolescent' age group (4.8 ± 1.9) *P *< 0.001 and the prevalence of a high (> 4 = median) number of risk alleles was less frequent in the 'elderly' (41.3%) than the adolescent subpopulation (56.3%, *P *< 0.001). The prevalence of a high (> 8 = median) number of risk alleles for DNA repair enzyme-coding genes was lower in the 'elderly' (37,3%) than the 'adolescent' subpopulation (45.6%, *P *= 0.017).

**Conclusions:**

These observations are consistent with the hypothesis that, in Flanders, the prevalence of at-risk alleles in genes involved in genotoxic effects decreases with age, suggesting that persons carrying a higher number of at risk alleles (especially in phase II xenobiotic-metabolizing or DNA repair genes) are at a higher risk of morbidity and mortality from chronic diseases. Our findings also suggest that, regarding risk of disease associated with low penetrance polymorphisms, multiple polymorphisms should be taken into account, rather than single ones.

## Background

Cancer and cardiovascular diseases are the main causes of severe morbidity and mortality in the developed world [1]. In western populations, morbidity and mortality rates of cardiovascular diseases, cancer and some other potentially fatal diseases increase rapidly between 15 and 65 years of age. According to Seer statistics, cancer incidence among non-hispanic whites in the USA in the year 2007 amounted to 19.2 per 100,000 persons below age 20, and to 874.9 per 100,000 persons aged 50 to 64 [2]. Based on rates from 2005-2007, 41.23% of white men and women born today in the USA will be diagnosed with cancer of all sites at some time during their lifetime [3]. Similar trends have been observed in Flanders, (an industrialized region in Belgium), which is the area under study in this report [4]. During youth and early adulthood, accidents and suicide are the main causes of death in Flanders. Between age 40 and 69 for women and age 50 and 79 for men, cancer is the main cause of death in Flanders. Later in life cardiovascular diseases are the main causes of mortality and severe morbidity [5]. Incidence of cancer steeply rises with age as carcinogenesis rests on the accumulation of several critical steps in time (mutations and also epigenetic events). Mortality from cardiovascular diseases shows an even more pronounced increase with age than cancer [5]. Without doubt, exposure to genotoxic carcinogens through smoking, consumption of alcohol and through diverse other life style related exposures or through environmental pollution importantly contributes to cancer risk [6]. Genotoxic substances such as those present in tobacco smoke and in polluted air also contribute to the causation of cardiovascular diseases [7]. Ironically, many of these genotoxic substances would not be health-threatening if they were not bioactivated through metabolism. Xenobiotic metabolism can be divided into two important phases [8]. During phase I, compounds can be activated by oxidation reactions resulting in the formation of highly reactive, electrophilic intermediates. In addition, these metabolic processes may contribute to the formation of reactive oxygen species (ROS) [9], which are inactivated by anti-oxidant defense mechanisms [10]. Electrophilic metabolites may be detoxified in phase II by the conjugation of endogenous ligands to the activated compounds, thereby increasing their hydrophilic nature and facilitating urinary excretion. An imbalance between oxidative metabolism and detoxification causes reactive intermediate products to accumulate, which may lead to DNA damage and eventually cause mutations. This may be counteracted by several DNA repair pathways which are capable of restoring DNA damage, thereby reducing mutation frequencies in genes involved in pathogenic processes including carcinogenesis and atheromatosis [11,12].

As carcinogenesis and atheromatosis ultimately result from an interaction between genetic and exogenous factors, variations between individuals in genes involved in the relevant pathogenic processes might explain to some degree why certain individuals within the general population are at a higher risk of disease, in spite of the fact that lifetime exposure to potentially hazardous compounds often does not greatly differ between individuals [13]. In 2008, we published a study conducted in a random selection of approximately 800 adolescent or elderly individuals, showing that part of the inter-individual differences in their reaction towards exposure to genotoxins can indeed be explained by genotypic differences [14]. It may consequently be hypothesized that in a random selection of individuals, able and willing to participate in a biomonitoring study and exposed to genotoxic substances, the prevalence of alleles, potentially affecting risk, will shift towards a higher frequency of the more 'protective' genotypes in 'elderly' individuals compared to adolescents, due to higher rates of morbidity and mortality among subjects with more unfavorable alleles.

Twin studies show that genetic differences account for about a quarter of the variance in adult human lifespan [15]. However, knowledge about genes that contribute to human longevity is limited. Few studies comparing prevalences of different genotypes between age groups exist, and contradicting results regarding the impact of certain genotypes on longevity were reported [16-19]. However, these studies focused mainly on only a few genotypes. Yet, it is very unlikely that one single low penetrance polymorphism is pivotal in carcinogenesis, atheromatosis and longevity. It is thus crucial to assess multiple genetic polymorphisms simultaneously, in order to obtain a better insight in human susceptibility, and thus longevity, in response to exposures to genotoxic substances.

Subjects invited to participate in this population-based biomonitoring study were randomly selected from the general population in the study area's comprising 22% of the surface area of Flanders and 20% of its population. Participation in the study implied being able to attend school (for adolescents) or to attend a consultation held by study nurses in a local community centre (for adults). Therefore, persons suffering from severe illness were unlikely to participate, although not formally excluded.

Available genotypes of 841 adolescents and elderly adults who participated in the Flemish Environment and Health Survey (FLEHS) were analyzed in an attempt to test the hypothesis that at-risk genotypes in relation to exposure to genotoxic substances decrease with age in a randomly selected population of persons able and willing to participate in a biomonitoring study. These adolescents [20] and adults [21] were shown to be internally exposed to: polycyclic aromatic hydrocarbons and benzene, long-time recognized human genotoxic carcinogens; lead and cadmium, carcinogenic [22] and indirectly genotoxic through disturbance of DNA repair and increasing oxidative stress [23-25]; and to the persistent organochlorines polychlorobiphenyls (PCBs), hexachlorobenzene and p,p'-dichlorodiphenyldichloroethylene (DDE), potentially increasing oxidative stress [26].

## Methods

### Study Population

In the Flemish Environment and Health Survey (FLEHS) a Stratified Clustered Multi-Stage Design was used as described for adults by De Coster et al. [21] and for adolescents by Schroijen et al. [20] to include 1,583 adults (775 men and 808 women) aged 50 to 65 (mean age: 57.6) and 1679 adolescents with a mean age of 14.9 years (range: 13.8-16.5) as a random sample of the population of the 8 areas under study, comprising 22% of the surface of Flanders and 20% of its population. The primary stage in the selection of adults was based on municipalities, and all contacted municipalities collaborated; concerning the adolescents, 42 of the 50 schools that were contacted collaborated. Table 1 shows the numbers of subjects selected using a Stratified Clustered Multi-Stage Design, the number of subjects participating in the FLEHS, and the number of subjects for whom genotyping was performed. The adolescents willing to participate had to attend a consultation with the study nurses in their respective schools where sampling of urine and blood, weighing, measuring and filling out of a self administered questionnaire took place. The adults willing to participate had to attend a consultation with the study nurses in a local community centre where sampling of urine and blood, weighing, measuring and filling out of a self administered questionnaire took place. So participants had to travel some distance in order to participate. Therefore, persons suffering from severe illness were unlikely to participate, although not formally excluded. Among the total of 1,583 adults participating in the FLEHS, DNA was available for analysis from a subset of 399 randomly selected individuals (176 females and 204 males). Among the 1679 adolescents, DNA was available for analysis from a subset of 442 randomly selected individuals (182 females and 260 males). A small proportion (N = 47; 11%) of the adolescents reported to smoke 7 ± 7 cigarettes per day and 63 adult individuals (16%) currently smoked 17 ± 10 cigarettes per day. Individuals who reported to have an ethnic background other than Caucasian, were excluded from analysis, because the prevalence of certain genotypes may differ between races [27]. Further inclusion criteria were living in Flanders for more than 5 years, and being able to complete questionnaires in Dutch. The study was approved by the ethical committee of the University of Antwerp, and every individual signed an informed consent prior to sample collection.

**Table 1:** Numbers of persons selected, participating in the Flesh and for whom genotyping was performed

	Invited	Participated (%)	Genotyped^1 ^(%)
Adolescents	2870	1679 (58.5%)	442 (15.4%)

Adults	4386	1583 (36.1%)	399 (9.1%)

^1^Randomly selected among the participants.

### White blood cell and DNA isolation

Immediately after blood collection, white blood cells were isolated by lysing erythrocytes using standard procedures and total white blood cells were stored as cell pellets at -80°C until DNA isolation. DNA was isolated in 96 wells plates using the Invisorb^® ^Blood Midi HTS 96 Kit/C (Invitek, Berlin, Germany), and DNA was subsequently stored in 96 wells plates at -20°C prior to genotyping at a concentration between 40 and 100 ng/μl.

### Selection of Polymorphisms and Genotyping

28 Low penetrance polymorphisms in 19 genes were selected. This selection was based on the involvement of the genes in processes relevant to genotoxicity thereby focusing on the commonly studied susceptibility genes in xenobiotic metabolism, oxidative stress defense and DNA repair (respectively 13, 6 and 9 polymorphisms, Table 2). Polymorphisms were selected in function of affecting the phenotype and having a minimum prevalence of 5%. Moreover, polymorphisms which could not clearly be categorized *a priori *as leading to an 'increased risk' or a 'protective effect' based on available literature data (see *statistical analysis*), were excluded from the analysis.

**Table 2:** Overview of the selected SNPs, their effect on enzyme function and definition of risk alleles

Polymorphism			Expected effect on enzymatic function	Putuative Risk Allele	Risk Allele leading to
*Biotransformation Phase I*					

*CYP1A2*	*A > C-154*	*(1)*	Higher inducibility	*-154****C***	
*CYP1A1*	*T > C3801*	*(4)*		*3801****C***	
	*I462V*	*(4)*		*462****V***	Increased formation of reactive metabolites (phase I enzymes)
	*T461N*	*(4)*	Higher enzyme activity	*461****N***	
*CYP2E1*	*G > T-70*	*(4)*		*-70****T***	
*CYP3A4*	*A > G-391*	*(4)*		*-391****G***	

*Biotransformation Phase II*					

*GSTM1*	*Del*	*(1)*	Deletion, no enzyme activity	***Del***	
*GSTT1*	*Del*	*(1)*		***Del***	
*GSTP1*	*I105V*	*(1)*		*105****V***	Decreased detoxification of reactive metabolites (phase II enzymes)
	*A114V*	*(1)*		*114****V***	
*NAT2*	*I114T*	*(1)*	Decreased enzyme activity	*114****T***	
	*R197Q*	*(1)*		*197****Q***	
	*G286E*	*(1)*		*286****E***	

*DNA Repair*					

*BRCA2*	*5'UTR (A-26G)*	*(2)*	Modulated expression	***A****-26*	
	*N372H*	*(2)*	Decreased enzyme activity	*372****H***	Decreased DNA repair
*XRCC1*	*R194W*	*(2)*	Increased enzyme activity	***R****194*	
	*R280H*	*(2)*	Decreased enzyme activity	*280****H***	
					
*XRCC3*	*T241M*	*(2)*		*241****M***	
*XPD*	*K751Q*	*(2)*		*751****Q***	
	*R156R*	*(2)*	Decreased enzyme activity	*156****R***	Decreased DNA repair
*APE1*	*D148G*	*(3)*		*148****G***	
*OGG1*	*S326C*	*(2)*		*326****C***	

*Oxidative Stress*					

*mnSOD*	*V16A*	*(3)*	Decreased enzyme activity	*16****A***	
*CAT*	*C > T-262*	*(3)*	Lower catalase activity	*-262****T***	Decreased oxidative stress defense
*NQO1*	*P187S*	*(3)*	Decreased enzyme activity	*187****S***	
	*R139W*	*(3)*		*139****W***	
*MPO*	*G > A-463*	*(4)*	Higher enzyme activity	***G****-463*	Higher production of free radicals
*GPX1*	*P198L*	*(3)*	Less efficient final GSH peroxidaxe complex	*198****L***	Impaired GSH metabolism; higher oxidative stress

(1)-(4): different multiplex PCRs (See Materials and Methods)

Genotyping was performed using the SNaPShot multiplex genotyping method described earlier [28]. Genotypes were determined in 4 different multiplex reactions (Table 2). For each multiplex PCR reaction, *Tm *was optimized: 56°C for multiplex 1, 60°C for multiplex 2, 57°C for multiplex 3 and 60°C for multiplex 4. Each of the multiplex PCR products was subsequently genotyped in a corresponding multiplex SBE reaction and analyzed on an ABI Prism^® ^3100 genetic analyzer using Genescan™ Analysis software (Version 3.7).

### Statistical Analysis

Risk alleles were identified based on expected phenotypic effects of the polymorphisms (Table 2). Genotypes were subsequently coded based on the number of risk alleles: homozygous carriers of alleles conferring an increase in risk were coded 2, carriers of 2 alleles with a putative protective health effect were coded 0 and heterozygous genotypes were coded 1. In case of GSTT1 and GSTM1, wild-types were coded 0 and deletions were coded 2; heterozygous individuals for these could not be identified with the SNaPshot procedure. For each individual, a sum of risk alleles was computed for all polymorphisms taken together and also for the processes of biotransformation, biotransformation phase I (CYP450s), biotransformation phase II (GSTs and NATs), oxidative stress and DNA repair separately. Differences between the two age groups concerning these sums of risk alleles were initially examined using the Mann-Whitney U test. Additionally, both for adolescents and adults, and for each of the biological processes under consideration, we determined the number of persons who showed more than the median number (based on the whole study population) of risk alleles. These persons were considered to carry a "high" number of risk alleles, whereas the remaining were considered to carry a "low" number of risk alleles. The prevalence of these "low" and "high" sums of risk alleles for each biological pathway were compared between the two age-groups by Chi-Square analysis using a 2 × 2 cross table. The number of persons included is large enough to detect a difference of 10% in the prevalence of a "high" number of risk alleles at an alpha error level of 5% with a statistical power of 82.7%. All statistical analyses were conducted using SPSS for windows, version 13.0. Results are presented as mean ± standard deviations.

## Results

In total, 23,548 genotypes were determined. Table 3 shows, per polymorphism, the numbers and percentages of adolescents and "elderly" with risk alleles. The theoretical maximum sum of risk alleles present in an individual, computed from this selection of polymorphisms, is: 28 polymorphisms genotyped per individual * 2 putative risk alleles = 56 risk alleles. The actual mean of the individual sum of risk alleles was 17.3 **± **3.0, with a range from 8 to 27 risk alleles. The mean sum of risk alleles for all investigated polymorphisms was found to be significantly lower in the 'elderly' (17.0 **± **2.9, range 8 to 25) compared to the adolescent (17.6 **± **3.1, range 10 to 27) population (*P *= 0.002). Nor in the adolescent nor in the "elderly" age group the results were affected by gender or smoking. In addition, subgroups of either a "low" or a "high" sum of risk alleles were defined, the cut-off point being determined by the median sum of risk alleles in the whole population, namely 17 risk alleles. The prevalence of a "high" sum of risk alleles (> 17 risk alleles) was significantly less frequent in the 'elderly' (40.6%) as compared to the adolescent (51.4%) subpopulation (*P *= 0.002, Chi-Square analysis) (see Table 4). Subsequently, differences in prevalence of risk alleles between the two age groups were examined for different biological processes (biotransformation, Phase I enzymes, phase II enzymes DNA repair and oxidative stress) separately.

**Table 3:** Percentages of adolescents and "elderly" persons with risk alleles

Gene	Risk Allele	Adolescents % with 1 risk allele	Elderly % with 1 risk allele	Adolescents % with 2 risk alleles	Elderly % with 2 risk alleles	Adolescents mean number of risk alleles	Elderly mean number of risk alleles
Biotransformation Phase I							

CYP1A2	-154C	32.3	36.8	7.3	7.0	0,468	0,509

CYP1A1	3801C	15.1	21.1	2.3	3.3	0,197	0,276

CYP1A1	462V	6.9	6.3%	0.0	0.0	0,069	0,063

CYP1A1	461N	7.6	7.8	0.5	0.0	0,085	0,078

CYP2E1	-70T	6.0	8.8	0.0	0.5	0,060	0,098

CYP3A4	-391G	6.0	5.8	1.1	0.5	0,083	0,068

Biotransformation Phase II							

GSTM1	Del	n.a.^1^	n.a.^1^	56.6	53.4	1,136	1,068

GSTT1	Del	n.a.^1^	n.a.^1^	18.4	14.3	0,367	0,286

GSTP1	105V	52.3	47.4	16.8	10.5	0,859	0,684

GSTP1	114V	12.5	14.5	2.5	1.0	0,175	0,165

NAT2	114T	49.9	49.6	30.6	19.8	1,113	0,892

NAT2	197Q	39.9	42.9	8.8	6.5	0,576	0,559

NAT2	286E	3.9	5.5	0.0	0.3	0,039	0,060

DNA Repair							

BRCA2	A-26	29.9	38.2	3.7	6.1	0,375	0,504

BRCA2	372H	46.2	41.5	9.4	10.6	0,657	0,628

XRCC1	R194	11.0	12.7	0.5	0.8	0,120	0,142

XRCC1	280H	9.7	9.4	0.5	0.3	0,108	0,099

XRCC3	241M	49.8	44.1	16.4	19.5	0,829	0,830

XPD	751Q	42.8	49.9	14.5	11.1	0,720	0,722

XPD	156R	43.7	51.9	24.6	19.7	0,933	0,914

APE1	148G	48.3	47.5	25.2	27.9	0,989	1,033

OGG1	326C	37.2	35.9	7.1	6.6	0,517	0,491

Oxidative Stresss							

mnSOD	16A	51.7	51.3	22.4	24.1	0,968	0,995

CAT	-262T	30.0	31.7	9.8	8.8	0,499	0,494

NQO1	187S	13.3	5.3	0.2	0.3	0,140	0,058

NQO1	139W	31.1	31.4	4.6	4.3	0,405	0,399

MPO	G-463	32.0	36.8	4.6	4.0	0,411	0,449

GPX1	198L	41.6	43.2	7.6	8.8	0,570	0,608

^1^Not available

**Table 4:** Numbers and percentage of adolescents and "elderly" persons with low versus high numbers of risk alleles

		**Low sum of risk alleles**^**1**^	**High sum of risk alleles**^**1**^	Chi-Square
**Total**	**Adolescents**	**203 (48.6%)**	**215 (51.4%)**	**p = 0.002**
	
	**Elderly**	**215 (59.4%)**	**147 (40.6%)**	

**Biotransformation**	**Adolescents**	**196 (45.7%)**	**233 (54.3%)**	**p = 0.005**
	
	**Elderly**	**204 (55.6%)**	**163 (44.4%)**	

**Phase I**	**Adolescents**	**321 (73.8%)**	**114 (26.2%)**	**p = 0.223**
	
	**Elderly**	**258 (69.9%)**	**111 (30.1%)**	

**Phase II**	**Adolescents**	**190 (43.7%)**	**245 (56.3%)**	**p < 0.001**
	
	**Elderly**	**233 (58.7%)**	**164 (41.3%)**	

**Oxidative Stress**	**Adolescents**	**256 (59.5%)**	**174 (40.5%)**	**p = 0.348**
	
	**Elderly**	**249 (62.7%)**	**148 (37.3%)**	

**DNA Repair**	**Adolescents**	**233 (54.4%)**	**195 (45.6%)**	**p = 0.017**
	
	**Elderly**	**247 (62.7%)**	**147 (37.3%)**	

^1^The cut-off point between low and high sum of risk alleles was based on the median number of risk alleles for each specific pathway. A high number of risk alleles was defined as: Total: > 17 risk alleles; Biotransformation:> 5 risk alleles; Phase I: > 1 risk allele; Phase II: > 4 risk alleles; Oxidative stress: > 4 risk alleles; DNA repair > 8 risk alleles.

### Biotransformation Pathway

**Figure 1:**
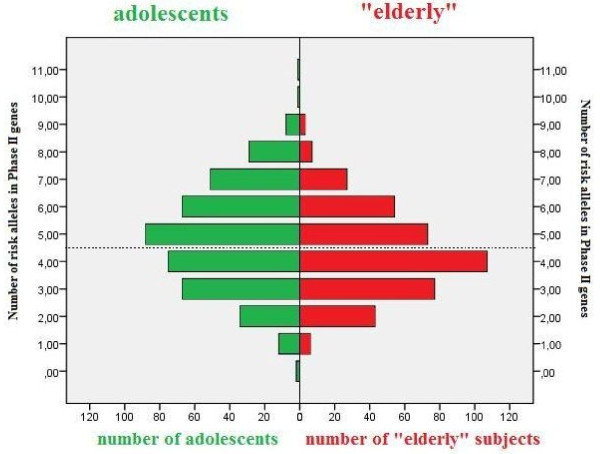
**Numbers of risk alleles in Phase II genes among the adolescents and the 'elderly'**. The dashed line represents the cut-off point between the relatively low (≤ 4) and high (> 4) sum of risk alleles (see methods). Compared to the adolescents there are less "elderly" persons with five to eleven risk alleles in phase II genes (p < 0.001). Moreover, the mean number of risk alleles is significantly lower in the 'elderly' population compared to the adolescent population (*P *< 0.001). As can be seen from the figure, among the "elderly" the group with 4 at risk alleles counts the most subjects, more then hundred (107), whereas among the adolescents the group with 5 at risk alleles counts the most subjects, almost 90 (88).

For all polymorphisms in biotransformation-related genes, the mean number of risk alleles was significantly lower in the 'elderly' age group (5.4 ± 1.9) as compared to the adolescent age group (5.8 **± **2.2, *P *= 0.003). Again, a cut-off sum of risk alleles in biotransformation enzymes was assessed based on the median value, namely 5. The presence of a relatively "high" sum of risk alleles in all selected biotransformation enzymes (> 5 risk alleles) was less frequent in the 'elderly' (44.4%) as compared to the adolescent population (54.3%, *P *= 0.005) (Table 4). The sum of risk alleles coding for biotransformation enzymes, was further subdivided into those coding for enzymes involved in bio-activation (phase I) and in detoxification (phase II) reactions. For phase II enzymes, the mean number of risk alleles was significantly lower in the 'elderly' (4.3 ± 1.6 risk alleles) as compared to the adolescent age group (4.8 ± 1.9 risk alleles, *P *< 0.001, Figure 1). Comparison of the prevalence of a "high" sum of risk alleles (cut off at 4 risk alleles, the median value) in phase II enzymes-coding genes showed a lower frequency of "high" sums of risk alleles in the 'elderly' (41.3%) as compared to the adolescent age group (56.3%, *P *< 0.001, Table 4). The number of risk alleles in genes coding for phase I enzymes was similar in the two age groups, with a mean of (1.1 ± 1.0) among the "elderly" and (1.0 ± 1.0) among the adolescents.

### DNA Repair pathway

**Figure 2:**
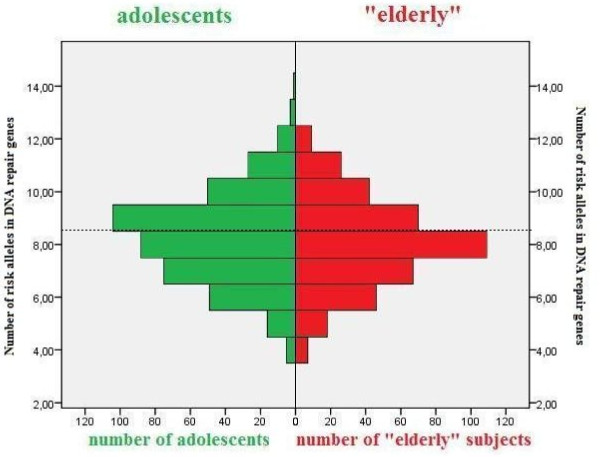
**Numbers of risk alleles in DNA repair genes among the adolescents and the 'elderly'**. The dashed line represents the cut-off point between the relatively low (≤8) and high (> 8) sum of risk alleles (see methods). The number of persons with nine to fourteen at-risk alleles is smaller among the elderly (p = 0.017). As can be seen from the figure, among the "elderly" the group with 8 at risk alleles counts the most subjects, more then hundred (109), whereas among the adolescents the group with 9 at risk alleles counts the most subjects, more the hundred (104).

For DNA repair enzymes, the mean number of risk alleles was slightly lower in the 'elderly' (8.07 risk alleles) as compared to the adolescent age group (8.25 risk alleles) but the difference was not statistically significant (*p = 0.15*). Comparison of the prevalence of a "high" sum of risk alleles (cut off at the median value of 8 risk alleles) for DNA repair enzyme-coding genes showed a lower frequency of "high" sums of risk alleles in the 'elderly' (37,3%) as compared to the adolescent age group (45.6%, *P *= 0.017, Table 4, Figure 2).

### Oxidative Stress pathway

The number of risk alleles in genes coding for oxidative stress-related genes was similar in the two age groups, with a slightly lower mean number of risk alleles among the "elderly" (4.1 ± 1.4) than among the adolescents (4.2 ± 1.4) (p = 0.58 ). A "high" number of risk alleles in oxidative stress-related genes was defined as > 4 (median value = 4). The presence of a "high" number of risk alleles was slightly less frequent in the "elderly" (37.3) as compared to the adolescents (40.5) (Table 4), but this difference was not statistically significant (p = 0.35).

## Discussion

We hypothesized that, in Flanders (an industrialized region in Belgium), the prevalence of an at-risk genotype -associated with a higher sensitivity to genotoxic agents- decreases with age due to morbidity and mortality resulting from chronic diseases. In our study, the prevalence of at risk genotypes (all genes considered taken together) in the elderly population was indeed observed to be lower than in the adolescent population. For the different biological processes taken separately, a statistically significant lower prevalence in the elderly was observed, in particular for phase II biotransformation genes. Among the elderly, the prevalence of a high number of unfavorable polymorphisms in DNA repair genes was also significantly lower. For genes involved in oxidative stress, a similar but statistically non-significant trend was observed. Our results suggest that Flemish residents carrying more unfavorable genetic traits related to genotoxic effects were more likely to be severely ill (so as to avoid participation in a biomonitoring study, participation for which travelling some distance was required) or to have died before age 50 to 65.

The relation between genetic traits and longevity is very complex and might well differ in function of the time interval (e.g. old age versus very old age) [18,29]. Nevertheless, an association was found between polymorphisms in certain genes and longevity [30,31]. For instance, in an ethnically homogeneous population, different age groups showed differences in the prevalence of certain polymorphisms in interleukin genes [32]. In particular, polymorphisms in genes related to inflammation, oxidative stress [32-34] and mitochondrial DNA [35] were found to be associated with differences in longevity. Although there are some contradictory reports in the literature [36,37] and although paradoxical effects were reported [18], a series of observations indicate that genetic traits in phase II enzymes, expected on mechanistic terms to increase risk, are indeed associated in humans with an increase in risk of cancer [19,38-43] or coronary heart disease [44,45]. In a cohort of not more than 354 persons Kayaalti et al. [46] detected an association of the interleukin-6 (IL-6) 174 G/C promoter region and MT2A -5 A/G core promoter region single nucleotide polymorphisms (SNPs) with longevity in the Turkish population.

Is it plausible to hypothesize that persons, who carry genetic traits which confer in some way an increased sensitivity to the pathogenic effects of genotoxic agents, are more likely to be severely ill or to die from disease before reaching the age of 50 to 65 years? The answer is probably yes, because, firstly, the exposure to genotoxic agents such as fine particles in polluted air, is associated with a decrease in life expectancy [47]. Secondly, it has also been shown that persons carrying certain genetic traits related to DNA repair, oxidative stress or metabolism of genotoxic substances, experience an increased risk of morbidity and mortality when exposed to genotoxic substances [48-50].

It has been established beyond doubt that the general population in a heavily industrialized region such as Flanders has an internal exposure to heavy metals and genotoxic or receptor binding pollutants at a level associated with multiple biological and health effects [20,21,51-53]. Both the adolescent and the adult participants in our FLEHS biomonitoring study appeared internally exposed to directly genotoxic agents such as polycyclic aromatic hydrocarbons, benzene and toluene, to heavy metals which may indirectly induce genotoxic effects, and to receptor-binding substances such as PCBs, hexachlorobenzene and DDE known to induce, at least under some circumstances, oxidative stress. Many of these pollutants are substrates for phase II detoxification enzymes [54]. All in all, it seems plausible to hypothesize that unfavorable alleles of genes involved in avoidance of genotoxic effects and in particular of genes involved in phase II detoxification are indeed associated with early severe morbidity and early mortality. Also, it is probable that the morbidity and mortality of persons carrying a high number of unfavorable genetic traits will differ more from morbidity and mortality of persons at younger age (under age 65) than at very old age, when morbidity and mortality are high among all persons.

Our findings regarding phase II genes are consistent with results from several case-control studies which have previously reported an association between polymorphisms in *NAT, GSTM *and *GST *and various types of cancer [19,38-43,55,56] or coronary heart disease [44,45]. It is remarkable that in our study the suggested negative association between unfavorable genetic traits and reaching age 50-65 without severe morbidity was most pronounced in relation with phase II enzymes, whereas it was less pronounced in relation to DNA repair genes. Interestingly, Laczmanska et al. [57] found that polymorphisms in genes encoding for xenobiotic metabolizing enzymes, had a greater influence on the genotoxic effect of diepoxybutane than polymorphisms in genes encoding for DNA repair proteins, which underlines our findings.

For genes involved in oxidative stress, the distribution of "low" and "high" sums of risk alleles between both age groups showed the same trend with lower numbers of risk alleles in the elderly, but this difference was very small and far from statistically significant. Conceivably, our "elderly" population was not old enough to allow differences with adolescents in oxidative stress to be statistically significant.

The cross-sectional design of the current study and the rather small number of subjects involved have to be considered important limitations. Additional observations, including preferably a prospective study, are certainly needed to substantiate this particular hypothesis. Still, the investigated study population appeared large enough to detect statistically significant differences between the 2 age groups. In addition, the genetic heterogeneity in the study population can be expected to be limited, as all participating individuals were Dutch-speaking Caucasians selected within the same regions in Flanders, and gender differences between the sub-populations were absent.

## Conclusions

The findings in this study may endorse the hypothesis that the prevalence of a high number of at-risk alleles in genes involved in genotoxic effects, especially in phase II biotransformation genes, decreases with age in a randomly selected population, suggesting that persons carrying a higher number of at risk alleles (especially in phase II xenobiotic-metabolizing or DNA repair genes) are at a higher risk of morbidity and mortality from chronic diseases. Our findings, as those of Rotunno et al [58], also suggest that, regarding risk of disease associated with low penetrance polymorphisms, multiple polymorphisms should be taken into account, rather than single ones. These results further suggest that it is of importance to continue the debate about the relevance of inter-individual differences in susceptibility with regard to human health risk assessment.

## List of abbreviations

DDE: p,p'-dichlorodiphenyldichloroethylene; DNA: Desoxyribonucleic acid; FLEHS: Flemish Environment and Health Survey; GSTM: Glutathione S transferase M; GSTM1: Glutathione S transferase M1; GST: Glutathione S transferase; GSTT1: Glutathione S transferase T1; NAT: N-acetyl transferase; PCBs: polychlorobiphenyls; PCR: Polymerase chain reaction; SBE: Single base extension; SNPs: single nucleotide polymorphisms.

## Competing interests

The authors declare that they have no competing interests.

## Authors' contributions

HBK contributed to the conception of the study, to the multiplex PCR analyses and to the drafting of the manuscript. RWLG contributed to the statistical analysis. RWHG contributed to the multiplex PCR analyses. AMK developed the multiplex PCR method. GK played a major role in organizing the Flemish biomonitoring study. GS co-ordinated the Flemish biomonitoring study. WFB co-ordinated the Flemish Environment and Health Centre and played a major role in organizing the Flemish biomonitoring study. JPMG contributed to the interpretation of the data and to the discussion. JHMvD supervised the laboratory work, contributed to the interpretation of the data and to the discussion. JCSK contributed to the conception of the study, contributed to the interpretation of the data and to the finalising of the manuscript. NAvL played a major role in organizing the Flemish biomonitoring study, contributed to the interpretation of the data and wrote the final manuscript. All authors read and approved the final manuscript.
